# Single-step versus conventional injectable artesunate for severe malaria in children: an open label, non-inferiority randomized clinical trial, Democratic Republic of the Congo and United Republic of Tanzania

**DOI:** 10.2471/BLT.25.293309

**Published:** 2025-11-12

**Authors:** Samwel Gesase, Marie Onyamboko, Caterina Fanello, Omari Abdul, Daddy Kalala Kayembe, Sarah Benie Bakomba, Daniel TR Minja, Bejos Kifakiou Nzambiwishe, Pascal Epe Ekombolo, Anangisye Malabeja, Joyce R Mbwana, Jaqueline Deen, George Mtove, Bipin Adhikari, Mohamed Mapondela, Chiraporn Taya, Brian Mutinda, Naomi Waithira, John PA Lusingu, Lorenz von Seidlein, Mavuto Mukaka, Arjen M Dondorp, Thomas J Peto

**Affiliations:** a National Institute for Medical Research, Korogwe Research Laboratory, Tanga, United Republic of Tanzania.; b Kinshasa School of Public Health, University of Kinshasa, Kinshasa, Democratic Republic of the Congo.; c Mahidol Oxford Tropical Medicine Research Unit, Faculty of Tropical Medicine, Mahidol University, 420/6 Rajvithi Rd, Bangkok 10400, Thailand.; d Institute of Child Health and Human Development, University of the Philippines, Manila, Philippines.

## Abstract

**Objective:**

To determine time and cost differences between one- and two-step injectable artesunate formulations for treatment of severe malaria and compare their safety and treatment outcomes.

**Methods:**

We conducted an open-label randomized clinical trial at hospitals in Kinshasa, Democratic Republic of the Congo and Korogwe, United Republic of Tanzania in patients aged 3 months to 16 years with severe malaria. We randomly allocated patients to a new one-step injectable artesunate formulation or the conventional two-step formulation. After discharge, patients were followed for 4 weeks. The main outcomes evaluated were time and cost of administering treatment, and clinical and pharmacodynamic effects.

**Findings:**

Between 7 June 2022 and 11 August 2023, 200 patients were randomized (1:1) to either the one-step or two-step arm. Mean time to administer artesunate was 2 min 22 s (standard deviation, SD: 50 s) in the one-step arm and 3 min 41 s (SD: 95 s) in the two-step (*P*-value: < 0.0001). Mean cost of syringes and needles used per patient was 0.53 (SD: 0.13) United States dollars (US$) in the one-step arm versus US$ 0.84 (SD:  0.22) in the two-step (*P*-value: 0.0001). Parasite clearance half-lives were 2.1 h (SD: 0.9) in the one-step arm and 2.0 h (SD: 0.8) in the two-step (*P* -value: 0.173). Severe adverse events occurred in one patient in each arm (*P* -value: 1.000), while 242 and 229 ungraded adverse events occurred in the one- and two-step arms, respectively (*P* -value: 0.549).

**Conclusion:**

In children with severe malaria, one-step injectable artesunate was quicker and cheaper to administer and had equivalent safety and efficacy compared with the conventional formulation.

## Résumé

**Objectif:**

Déterminer les différences en termes de durée et de coût entre les formulations d’artésunate injectable en une et en deux étapes pour le traitement du paludisme grave et comparer leur innocuité et leurs résultats thérapeutiques.

**Méthodes:**

Nous avons mené un essai clinique randomisé ouvert dans des hôpitaux de Kinshasa (République démocratique du Congo) et de Korogwe (République-Unie de Tanzanie) auprès de patients âgés de 3 mois à 16 ans et atteints de paludisme grave. Nous avons randomisé les patients entre une nouvelle formulation d’artésunate injectable en une seule étape et la formulation conventionnelle en deux étapes. Après leur sortie de l’hôpital, les patients ont fait l’objet d’un suivi pendant 4 semaines. Les principaux résultats évalués étaient la durée et le coût de l’administration du traitement, ainsi que les effets cliniques et pharmacodynamiques.

**Résultats:**

Entre le 7 juin 2022 et le 11 août 2023, 200 patients ont été randomisés (selon un rapport 1:1) dans les groupes à une étape et à deux étapes. Le temps moyen d’administration de l’artésunate était de 2 min 22 s (écart-type (ET): 50 s) dans le groupe à une étape et de 3 min 41 s (ET: 95 s) dans le groupe à deux étapes (valeur P: < 0,0001). Le coût moyen des seringues et des aiguilles employées par patient était de 0,53 dollar américain (USD) (ET: 0,13) dans le groupe à une étape contre 0,84 USD (ET: 0,22) dans le groupe à deux étapes (valeur P: 0,0001). La demi-vie d’élimination du parasite était de 2,1 h (ET: 0,9) dans le groupe à une étape et de 2,0 h (ET: 0,8) dans le groupe à deux étapes (valeur P: 0,173). Des événements indésirables graves sont survenus chez un patient dans chaque groupe (valeur P: 1,000), tandis que 242 et 229 événements indésirables non classés sont survenus dans le groupe à une étape et dans le groupe à deux étapes, respectivement (valeur P: 0,549).

**Conclusion:**

Chez les enfants atteints de paludisme grave, l’artésunate injectable en une seule étape était plus rapide et moins coûteux à administrer et présentait une innocuité et une efficacité équivalentes à celles de la formulation conventionnelle.

## Resumen

**Objetivo:**

Determinar las diferencias en tiempo y costo entre las formulaciones de artesunato inyectable de uno y dos pasos para su preparación para el tratamiento del paludismo grave, y comparar su seguridad y resultados terapéuticos.

**Métodos:**

Se realizó un ensayo clínico aleatorizado, abierto, en hospitales de Kinshasa (República Democrática del Congo) y Korogwe (República Unida de Tanzania) en pacientes de entre 3 meses y 16 años con paludismo grave. Los pacientes se asignaron aleatoriamente a una nueva formulación de artesunato inyectable de un solo paso para preparación o a la formulación convencional de dos pasos para preparación. Tras el alta, los pacientes fueron seguidos durante cuatro semanas. Los principales desenlaces evaluados fueron el tiempo y el costo de administración del tratamiento, así como los efectos clínicos y farmacodinámicos.

**Resultados:**

Entre el 7 de junio de 2022 y el 11 de agosto de 2023 se aleatorizaron 200 pacientes (1:1) en los dos grupos de tratamiento. El tiempo medio de administración del artesunato fue de 2 minutos y 22 segundos (desviación estándar, DE: 50 s) en el grupo de un solo paso y de 3 minutos y 41 segundos (DE: 95 s) en el grupo de dos pasos (valor de *p*: <0,0001). El costo medio de jeringas y agujas utilizadas por paciente fue de 0,53 dólares estadounidenses (US$) (DE: 0,13) en el grupo de un solo paso y de US$ 0,84 (DE: 0,22) en el grupo de dos pasos (valor de *p*: 0,0001). Las semividas de eliminación del parásito fueron de 2,1 h (DE: 0,9) en el grupo de un solo paso y de 2,0 h (DE: 0,8) en el grupo de dos pasos (valor de *p*: 0,173). Se produjeron acontecimientos adversos graves en un paciente de cada grupo (valor de *p*: 1000), mientras que se registraron 242 y 229 acontecimientos adversos no clasificados en los grupos de uno y dos pasos, respectivamente (valor de *p*: 0,549).

**Conclusión:**

En los niños con paludismo grave, el artesunato inyectable de un solo paso para preparación fue más rápido y económico de administrar. Además, presentó una seguridad y eficacia equivalentes a las de la formulación convencional.

## ملخص

الغرض تحديد فروق التوقيت والتكلفة بين تركيبات الأرتيسونات القابلة للحقن أحادية الخطوة وثنائية الخطوة لعلاج الملاريا الحادة، ومقارنة سلامتها ونتائج علاجها.

الطريقة قمنا بإجراء تجربة إكلينيكية عشوائية مفتوحة في مستشفيات كينشاسا، بجمهورية الكونغو الديمقراطية، مدينة كوروجوي، وجمهورية تنزانيا المتحدة، على مرضى تتراوح أعمارهم بين 3 أشهر و16 عامًا مصابين بالملاريا الحادة. قمنا بتوزيع المرضى بشكل عشوائي على تركيبة أرتيسونات جديدة أحادية الخطوة قابلة للحقن، أو التركيبة التقليدية ثنائية الخطوة. بعد خروج المرضى من المستشفى، تمت متابعتهم لمدة 4 أسابيع. شملت النتائج الرئيسية التي تم تقييمها وقت وتكلفة إعطاء العلاج، والآثار الإكلينيكية والديناميكية الدوائية.

النتائج بين 7 يونيو/حزيران 2022، و11 أغسطس/آب 2023، تم توزيع 200 مريض عشوائيًا (1:1) على مجموعة العلاج، إما أحادية الخطوة، أو ثنائية الخطوة. بلغ متوسط وقت إعطاء الأرتيسونات دقيقتين و22 ثانية (الانحراف المعياري: 50 ثانية) في مجموعة العلاج أحادية الخطوة، و3 دقائق و41 ثانية (الانحراف المعياري: 95 ثانية) في مجموعة العلاج ثنائية الخطوة (قيمة نسبة الاحتمال: < 0.0001). بلغ متوسط تكلفة الحقن والإبر المستخدمة لكل مريض 0.53 (الانحراف المعياري: 0.13) دولار أمريكي في مجموعة العلاج أحادية الخطوة، مقابل 0.84 دولار أمريكي (الانحراف المعياري:  0.22) في مجموعة العلاج ثنائية الخطوة (قيمة نسبة الاحتمال: 0.0001). بلغ نصف عمر تصفية الطفيليات 2.1 ساعة (الانحراف المعياري: 0.9) في مجموعة العلاج أحادية الخطوة، و20 ساعة (الانحراف المعياري: 0.8) في مجموعة العلاج ثنائية الخطوة (قيمة نسبة الاحتمال: 0.173). ظهرت آثار جانبية شديدة لدى مريض واحد في كل مجموعة علاج (قيمة نسبة الاحتمال: 1.000)، بينما ظهر 242 و229 أثرًا جانبيًا غير مُصنَّف في مجموعتي العلاج أحادية الخطوة وثنائية الخطوة، على التوالي (قيمة نسبة الاحتمال: 0.549).

الاستنتاج في الأطفال المصابين بالملاريا الحادة، كانت الأرتيسونات أحادية الخطوة القابلة للحقن أسرع وأرخص في التعاطي، ولها سلامة وفعالية مكافئة مقارنةً بالتركيبة التقليدية.

## 摘要

**目的:**

旨在确定用于治疗严重疟疾的一步和两步注射用青蒿琥酯制剂在时间和成本方面的差异，并对比二者之间的安全性和疗效。

**方法:**

在刚果民主共和国金沙萨和坦桑尼亚联合共和国科罗圭的医院里，我们针对患有严重疟疾且年龄介于 3 个月至 16 岁之间的患者开展了一项开放性随机临床试验。我们以随机分配的方式安排患者接受全新的一步注射用青蒿琥酯制剂或常规两步注射用青蒿琥酯制剂。待患者出院后，再对其进行为期 4 周的跟踪随访。基于试验结果，我们主要评估了开展治疗所耗费的时间和成本以及临床效果和药效作用。

**结果:**

在 2022 年 6 月 7 日至 2023 年 8 月 11 日期间，我们将 200 名患者随机分配（按 1:1 的比例）至一步法试验组或两步法试验组。注射青蒿琥酯的平均时间分别为 2 分 22 秒【一步法试验组，标准偏差 (SD)：50 秒】和 3 分 41 秒（两步法试验组，SD：95 秒）（*P *值：< 0.0001）。每名患者使用注射器和针头所耗费的平均成本分别为 0.53 美元（一步法试验组，SD：0.13）和 0.84 美元（两步法试验组，SD：0.22）（*P* 值：0.0001）。寄生虫清除半衰期分别为 2.1 小时（一步法试验组，SD：0.9）和 2.0 小时（两步法试验组，SD：0.8）（*P *值：0.173）。两个试验组都出现了一例严重不良事件（*P *值：1.000），且两个组分别出现了 242 例（一步法试验组）和 229 例（两步法试验组）未分级不良事件（*P *值：0.549）。

**结论:**

针对一步和常规制剂的对比研究结果表明，治疗患有严重疟疾的儿童时，一步注射用青蒿琥酯的给药速度更快且成本更低，但其安全性和疗效却与常规制剂相当。

## Резюме

**Цель:**

Определить различия во времени и затратах для применения одно- или двухэтапной формы введения инъекционных препаратов артесуната при лечении тяжелой малярии у детей, а также сравнить безопасность и исходы лечения.

**Методы:**

Было проведено открытое рандомизированное клиническое исследование в больницах Киншасы (Демократическая Республика Конго) и Корогве (Объединенная Республика Танзания) среди пациентов с тяжелой малярией в возрасте от 3 месяцев до 16 лет. Пациентов случайным образом распределяли для получения новой одноэтапной инъекционной формы артесуната или стандартной двухэтапной формы. После выписки за пациентами велось контрольное наблюдение на протяжении 4 недель. Основными оцениваемыми исходами были время и затраты на предписанное лечение, а также клинические и фармакодинамические эффекты.

**Результаты:**

В период с 7 июня 2022 года до 11 августа 2023 года 200 пациентов были рандомизированы (1:1) в группу либо одноэтапной, либо двухэтапной формы применения препарата. Среднее время введения артесуната составило 2 минуты 22 секунды (стандартное отклонение, СО: 50 с) в группе одноэтапной формы препарата и 3 минуты 41 секунда (СО: 95 с) в группе двухэтапной формы препарата (*Р*-значение: < 0,0001). Средняя стоимость шприцев и игл из расчета на одного пациента составила 0,53 (СО: 0,13) доллара США (долл. США) в группе одноэтапной формы препарата и 0,84 долл. США (СО: 0,22) в группе двухэтапной формы препарата (*Р*-значение: 0,0001). Период полувыведения паразитов составил 2,1 часа (СО: 0,9) в группе одноэтапной формы препарата и 2,0 часа (СО: 0,8) в группе двухэтапной формы препарата (*Р*-значение: 0,173). Тяжелые нежелательные явления наблюдались у одного пациента в каждой из групп (*Р*-значение: 1,000), тогда как 242 и 229 нежелательных явлений неуказанной степени тяжести наблюдались соответственно в группах одноэтапной и двухэтапной формы введения препарата (*Р*-значение: 0,549).

**Вывод:**

В случае тяжелой малярии у детей одноэтапная форма инъекционного артесуната требовала меньше времени и затрат на введение, равно как имела безопасность и эффективность, эквивалентную стандартной лекарственной форме.

## Introduction

Severe malaria is a main cause of preventable childhood death in the World Health Organization (WHO) African Region.^1^^,^^2^ Outcomes depend on rapid access to health care and initiation of antimalarial treatment. Delaying treatment is potentially lethal.^3^ The African Region accounted for about 94% (233/249 million) of global cases of malaria in 2022 with four countries reporting half of all malaria deaths: Nigeria, Democratic Republic of the Congo, Niger and United Republic of Tanzania, in descending order of total deaths.^4^

Parenteral artesunate is the first-line treatment for severe malaria^5^ based on the results of two large trials in the African and South-East Asia regions comparing parenteral artesunate with quinine. In both trials, artesunate was associated with a significant reduction in mortality compared with quinine, a 35% (107/730 versus 164/731) relative reduction in deaths in mainly adult patients in South-East Asia and a 23% (230/2712 versus 297/2713) reduction in paediatric patients in Africa.^6^^,^^7^ No increase in severe sequelae was seen after treatment with parenteral artesunate. Injectable artesunate was also shown to be cost-effective.^8^ These findings resulted in the WHO recommendation to use parenteral artesunate as first-line antimalarial therapy for severe malaria in all endemic settings.^9^ Since 2011, this treatment change is estimated to have saved hundreds of thousands of lives.^10^

The current formulation of injectable artesunate requires a two-step process, comprising reconstitution of the artesunate hemisuccinate powder in a sodium bicarbonate solution followed by further dilution in 5% dextrose or normal saline.^11^ The preparation of the two-step formulation takes time, is prone to error and requires additional consumables, including sterile syringes. The current formulation requires gently shaking the reconstituted artesunate solution for 3 minutes to 5 minutes to ensure that it is completely dissolved. In addition, the final concentrations required for intravenous and intramuscular routes are different, creating another potential source of error.^12^ Errors in preparation can cause wastage of drugs, and misdosing can put critically ill patients at increased risk of harm. Previous studies have highlighted gaps in health workers’ knowledge of artesunate-based treatment of severe malaria, suggesting the need to simplify intravenous artesunate regimens.^13^^,^^14^

**Fig. 1:**
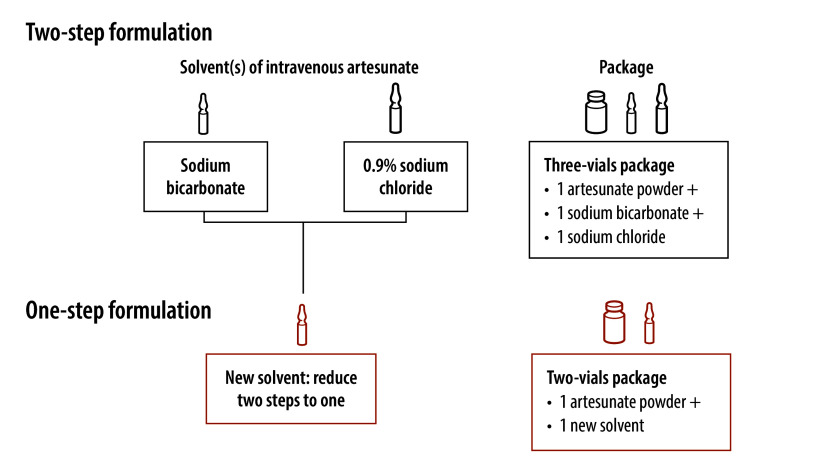
One- and two-step formulations of injectable artesunate

In a medical emergency such as severe malaria, delays in starting treatment can have life-threatening consequences. Severe malaria often develops in rural communities where hospital referral may take hours or days. The new reformulation of injectable artesunate requires a single-step reconstitution and could potentially be given at more peripheral levels of the health system. Bioequivalence studies in healthy volunteers showed matching exposures to artesunate and dihydroartemisinin whether administered intravenously or intramuscularly.^15^ The one-step formulation uses the same volume of solvent for intravenous and intramuscular injection, thus reducing the potential for confusion.^16^ In addition, the one-step formulation could reduce costs, because its administration may take less time and use fewer consumables (Fig. 1).

As new treatments for severe malaria could affect millions of vulnerable patients, our study aimed to obtain direct evidence of real-life use of the one-step artesunate formulation and to assess and mitigate against the risk of any unexpected differences in treatment effects. Specifically, we conducted a clinical trial to: (i) quantify the time and cost differences between the one- and two-step artesunate formulations; (ii) compare their safety and treatment outcomes; and (iii) identify any unanticipated issues.

## Methods

This open-label, individually randomized, non-inferiority trial compared the two artesunate formulations. We compared the speed and convenience of preparation, the costs of drug administration, parasite clearance time and adverse events. The intervention in both arms of the trial was artesunate given at the same dosage. We captured pharmacodynamic and clinical outcomes to exclude the possibility of unexpected differences in drug activity due to varying formulations.^8^

### Study design and participants

We conducted the study in Korogwe, United Republic of Tanzania and Kinshasa, Democratic Republic of the Congo, countries with a substantial burden of severe *Plasmodium falciparum* malaria. Patients aged 3 months to 16 years were eligible if they had clinically diagnosed severe *P. falciparum* malaria based on a positive malaria rapid diagnostic test or microscopically confirmed *P. falciparum* with > 350 000 parasites/µL. Clinical criteria for severe malaria were: impaired consciousness; prostration; failure to feed and drink without assistance; multiple convulsions; respiratory distress; circulatory collapse or shock; systolic blood pressure < 70 mmHg; clinical jaundice plus evidence of other vital organ dysfunction; haemoglobinuria; suspected pulmonary oedema; renal failure; spontaneous bleeding; repeated vomiting in the preceding 24 hours; or severe pallor with respiratory distress. Exclusion criteria were current participation in any other medical trial, body weight < 5 kg, known allergy to artemisinin derivatives, or parenteral treatment for severe malaria during the present illness. Treatment with an oral antimalarial or with a single dose of pre-referral rectal artesunate were not exclusion criteria. In both Democratic Republic of the Congo and United Republic of Tanzania, the cost of treatment was covered by the study. In routine practice, the cost of treatment for children younger than 5 years is covered by the governments of each country; even so, parents often need to purchase items such as consumables.

### Study procedures

We randomized eligible patients at each study site in a 1:1 ratio (in block sizes of 8, 10 and 12, to which study staff were blinded). The trial statistician at Mahidol Oxford Tropical Medicine Research Unit in Bangkok computer-generated the sequences and supervised preparation of randomization envelopes. Allocation was performed by opening the next sequentially numbered opaque envelope, which contained the study number and treatment. Once that envelope was opened, the patient was irrevocably enrolled. To minimize potential bias, staff performing laboratory investigations after enrolment were blind to treatment allocation.

Patients were admitted to the study ward and randomly allocated to either one-step or two-step injectable artesunate, both administered intravenously every 12 hours for a minimum of three doses. A member of the study team, usually a nurse, directly observed the nurses and doctors at the treatment centres administer the drug preparation. A senior doctor from each site trained and supervised the study staff. Artesunate dosing was according to WHO guidelines. When the patient was able to tolerate oral medication, injectable treatment was replaced with a full 3-day course of an artemisinin combination therapy (artemether–lumefantrine) to complete antimalarial treatment. The use of concomitant medications and treatment was unrestricted.

Patients remained in the study ward until parasite clearance and were advised to remain until completion of treatment. To determine asexual parasite clearance half-life, study staff took blood samples at hour 4, 8, 12 hours and thereafter at 6-hourly intervals until clearance on two consecutive smears examined after Giemsa-staining blood.^17^ After discharge, study staff assessed patients weekly as outpatients at day 7, 14, 21 and 28. In case of important unresolved sequelae at day 28, follow-up was extended for up to 12 months.^18^

A time and motion study recorded the time to prepare the artesunate solution for injection and to administer treatment, the number of actions performed to prepare treatment, and the consumables used.^19^ We estimated the cost of consumables based on local prices provided by the hospital pharmacy.

### Outcomes

The primary outcomes were time required to prepare the artesunate for injection and the cost of consumables, including the cost of syringes with needles. Exploratory outcomes were severe adverse and other adverse outcomes (graded according to Common Terminology Criteria for Adverse Events, version 5.0)^20^ as indicators of safety and fever clearance times, and time to tolerate oral medication as treatment outcomes (all stratified by study site). We assessed parasite clearance rates as a measure of the pharmacodynamic effect. We will report secondary outcomes of the study that explored feasibility and acceptability of the one-step formulation in a separate article.

### Statistical methods

We followed a pre-specified statistical analysis plan to report this trial. The original power calculations for the primary outcome assumed that conventional injectable artesunate requires 4 min to prepare for administration with a standard deviation (SD) of 2 min. Our non-inferiority margin for the new formulation was 1 min. With this non-inferiority margin, detecting non-inferiority with 90% power and with a one-sided *α* of 0.025 required inclusion of 170 participants. Allowing for a 15% loss to follow-up or withdrawal, we aimed to recruit 200 participants in the two arms combined. The study was not powered to produce statistically reliable disaggregated data by sex and/or gender and there was no *a priori* reason to expect any such difference. To compare outcomes between treatment groups, we calculated medians (interquartile range, IQR) and difference in means (95% confidence interval, CI). We used unpaired *t*-tests or the Wilcoxon rank-sum (Mann–Whitney) test as appropriate to evaluate differences in continuous data. We compared proportions with the Fisher exact test. We used the WorldWide Antimalarial Resistance Network calculator to determine parasite clearance half-lives.^21^ We used Stata, version 18 (StataCorp. LP, College Station, United States of America) for all analyses.

### Approvals and consent

The following ethics committees approved the study: National Health Research Ethics Sub-Committee, United Republic of Tanzania; Research Ethics Committee of the Kinshasa School of Public Health and the Ministry of Health of Democratic Republic of the Congo; and the Oxford University Tropical Research Ethics Committee, United Kingdom of Great Britain and Northern Ireland. Oxford University was the study sponsor. The Mahidol-Oxford Tropical Medicine Research Unit monitored the study conduct. A parent or guardian gave written informed consent, witnessed when necessary, according to good clinical practice. Additionally, Tanzanian patients aged 12 years or older gave their own informed assent. We had all study documents translated into local language as appropriate. A data and safety monitoring board met before the trial and twice during the recruitment period. We registered the trial with ClinicalTrials.gov (NCT05140278). 

## Results

Between 7 June 2022, and 11 August 2023, we assessed 230 patients for eligibility and enrolled and randomized 200 of these patients in the study.

### Baseline characteristics

**Fig. 2:**
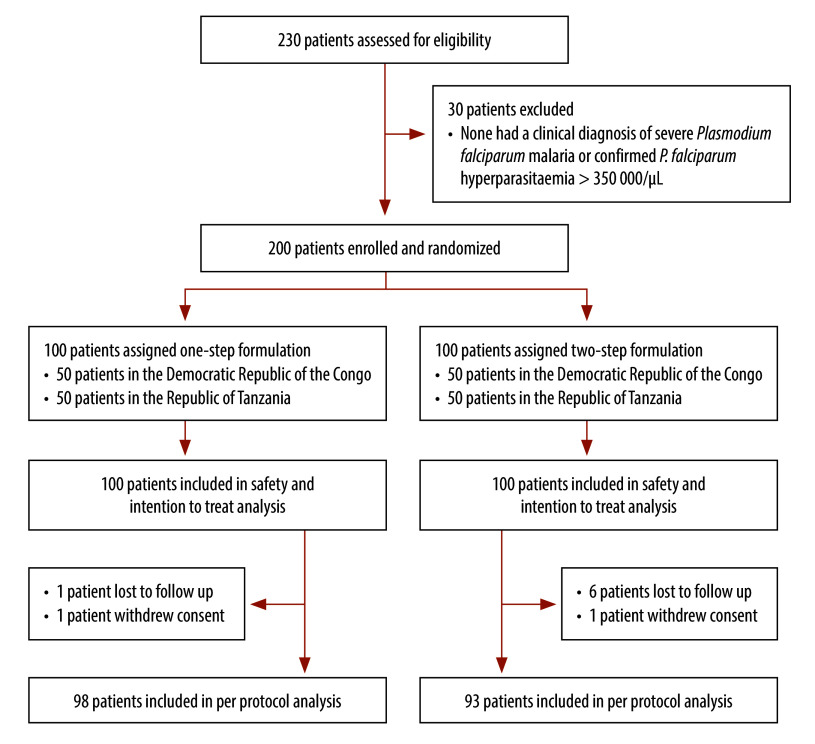
Flowchart of selection of study participants for the comparison between one- and two-step injectable artesunate formulations, Democratic Republic of the Congo and United Republic of Tanzania, 2022–2023

Fig. 2 summarizes the selection and randomization of study participants. Of the 200 participants, 95 (48%) were male. The median age was 4.2 years (IQR: 2.3–7.8) and the geometric mean of the baseline parasitaemia (at hour 0) was 67 513 parasites/µL (IQR: 12 952‒222 529). The mean weight was 17.0 kg (SD: 8.6) and mean haemoglobin was 6.1 mmol/L (SD: 1.5). The baseline characteristics of the patients by treatment arm are summarized in Table 1. The characteristics were well balanced across the two treatment arms.

**Table 1:** Baseline patient characteristics, by formulation used, Democratic Republic of the Congo and United Republic of Tanzania, 2022–2023

Characteristic	One-step formulation (*n* = 100)	Two-step formulation (*n* = 100)	Total (*n* = 200)
Age in years, median (IQR)	4.0 (2.1–7.0)	4.7 (2.5–8.0)	4.2 (2.3–7.8)
Male sex, no. (%)	46 (46)	49 (49)	95 (48)
Weight in kg, mean (SD)	16.5 (8.3)	17.5 (8.8)	17.0 (8.6)
Height in cm, mean (SD)	102.9 (23.2)	105.2 (24.9)	104.0 (24.1)
Temperature in °C, mean (SD)	38.1 (1.1)	37.8 (1.0)	38.0 (1.1)
Haemoglobin in mmol/L, mean (SD)	6.0 (1.6)	6.2 (1.3)	6.1 (1.5)
Parasite load per µL, geometric mean (IQR)	80 629 (12 623–244 568)	56 973 (12 952–181 856)	67 513 (12 952–222 529)
Impaired consciousness or unrousable coma, no. (%)	1 (1)	1 (1)	2 (1)
Prostration, no. (%)	75 (75)	76 (76)	151 (76)
Failure to feed and drink without assistance, no. (%)	44 (44)	38 (38)	82 (41)
Multiple convulsions, no. (%)	13 (13)	11 (11)	24 (12)
Deep breathing, respiratory distress, no. (%)	28 (28)	29 (29)	57 (28)
Circulatory collapse or shock (systolic blood pressure < 70 mmHg), no. (%)	2 (2)	1 (1)	3 (2)
Clinical jaundice plus evidence of other vital organ dysfunction, no. (%)	2 (2)	1 (1)	3 (2)
Haemoglobinuria, no. (%)	0 (0)	1 (1)	1 (0.5)
Suspected pulmonary oedema, no. (%)	1 (1)	1 (1)	2 (1)
Renal failure (< 20 mL urine/hour), no. (%)	0 (0)	1 (1)	1 (0.5)
Spontaneous bleeding and/or disseminated intravascular coagulation, no. (%)	0 (0)	1 (1)	1 (0.5)
Several episodes of vomiting in the preceding 24 hours, no. (%)	63 (63)	59 (59)	122 (61)
Severe pallor with respiratory distress or haematocrit < 15% or haemoglobin < 5.0 g/dL, no. (%)	9 (9)	9 (9)	18 (9)

IQR: interquartile range; SD: standard deviation.

### Convenience, costs and speed

We compared time to administration of treatment and costs of administration at the health facility between the one- and two-step formulations, using time-and-motion methods (Table 2).

**Table 2:** Drug preparation time, and number and cost of injection supplies used, by formulation used, Democratic Republic of the Congo and United Republic of Tanzania, 2022–2023

Variable	Mean (SD)		Difference in means (95% CI)
	One-step formulation (*n* = 100)	Two-step formulation (*n* = 100)	
**Time from opening box to completed syringe preparation^a^ in min:s**			
Overall	2:22 (0:50)	3:41 (1:35)	−1:19 (−1:32 to −1:07)
Democratic Republic of the Congo	2:30 (0:36)	4:11 (1:31)	−1:31 (−1:47 to −1:16)
United Republic of Tanzania	2:13 (1:00)	3:22 (1:35)	−1:08 (−1:27 to −1:03)
**Syringes and needles^b^ used per patient, no.**			
Overall	6 (2)	9 (3)	−3 (−4 to −2)
Democratic Republic of the Congo	6 (2)	9 (1)	−3 (−3 to −2)
United Republic of Tanzania	6 (2)	9 (4)	−3 (−4 to −2)
**Cost of syringes and needles^c^ per patient in US$**			
Overall	0.53 (0.13)	0.84 (0.22)	–0.31 (–0.35 to –0.26)
Democratic Republic of the Congo	0.50 (0.13)	0.78 (0.19)	–0.28 (–0.35 to –0.22)
United Republic of Tanzania	0.57 (0.12)	0.90 (0.23)	–0.33 (–0.40 to –0.26)

CI: confidence interval; SD: standard deviation; US$: United States dollars.

^a^ At all recorded times.

^b^ Syringes 5 mL and 10 mL.

^c^ Calculated from average cost of consumables used and wastage of the drug.

The mean time from opening the study box to completed preparation of the syringe for all recorded times was 2 min 22 s (SD: 50 s) in the one-step arm and 3 min 41 s (SD: 1 min 35 s) in the two-step arm. The difference in means was −1 min 19 s (95% CI: −1 min 32 s to −1 min 7 s), a 36% (79/221) reduction with the one-step formulation. The mean total number of syringes used (syringes 5 mL and 10 mL, to accommodate different sizes of children) per patient, at all recorded times was 6 pieces (SD: 2) in the one-step arm versus 9 pieces (SD: 3) in the two-step arm; difference in means −3 (95% CI: −4 to −2). The mean total cost of syringes and needles used (syringes 5 mL and 10 mL) per patient in United States dollars (US$), at all recorded times, was US$ 0.53 (SD: 0.13) in the one-step arm versus US$ 0.84 (SD: 0.22) in the two-step arm. The difference in means was −US$ 0.31 (95% CI: US$ −0.35 to US$ −0.26), a 37% (31/84) reduction in costs with the one-step formulation.

### Fever and parasite clearance

**Fig. 3:**
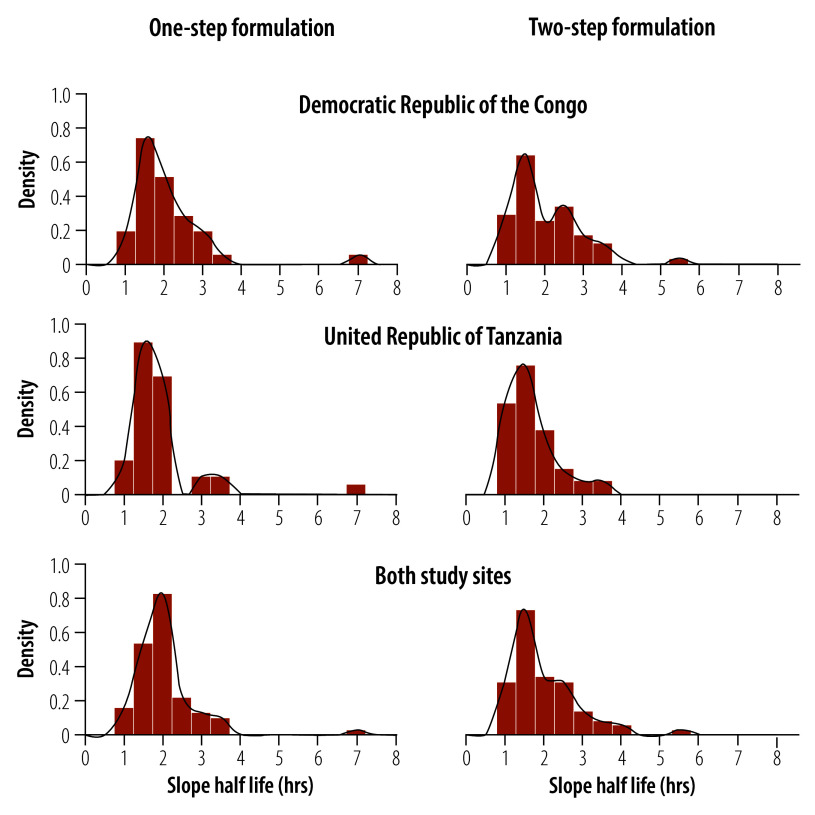
Parasite clearance half-life distribution for the two formulations and countries, Democratic Republic of the Congo and United Republic of Tanzania, 2022–2023

The mean time to fever clearance was 13.5 h (SD: 13.8) in the one-step arm, compared with 11.2 h (SD: 12.5) in the two-step arm (*P*-value: 0.190). The mean time to be able to tolerate oral medication was 32.6 h (SD: 5.0) in the one-step arm compared with 31.6 h (SD: 3.9) in the two-step arm (*P*-value: 0.369). Parasite clearance half-lives and proportions of patients with parasitaemia persisting to day 3 were similar in the two treatment arms (Table 3 and Fig. 3). With the one-step formulation, the mean time to parasite clearance was 2.1 h (SD: 0.9) compared with 2.0 h (SD: 0.8) for the two-step arm (*P*-value: 0.173). The proportion of patients with parasites on day 3 was 1% (1/100) in the one-step arm compared with 0% (0/100) in the two-step arm (*P*-value: 1.000; Table 3).

**Table 3:** Clinical recovery, by formulation used, Democratic Republic of the Congo and United Republic of Tanzania, 2022–2023

Variable	One-step formulation	Two-step formulation	Both groups	*P* ^a^
**Fever clearance in h, mean (SD)^b^**				
Democratic Republic of the Congo	8.2 (13.0)	6.2 (11.1)	7.2 (12.1)	0.341
United Republic of Tanzania	18.8 (12.7)	16.2 (11.8)	17.5 (12.3)	0.292
All study sites	13.5 (13.8)	11.2 (12.5)	12.4 (13.2)	0.190
**Positive day 3 blood smear, no./*n*(%)^c^**				
Democratic Republic of the Congo	1/50 (2)	0/50 (0)	1/100 (1)	1.000^d^
United Republic of Tanzania	0/50 (0)	0/50 (0)	0/100(0)	NA
All study sites	1/100 (1)	0/100 (0)	1/200 (1)	1.000^d^
**Time to tolerate oral medication in h, mean (SD)^e^**				
Democratic Republic of the Congo	32.7 (5.2)	32.0 (0.6)	32.4 (3.7)	0.979
United Republic of Tanzania	32.6 (5.0)	31.2 (5.5)	31.9 (5.2)	0.349
All study sites	32.6 (5.0)	31.6 (3.9)	32.1 (4.5)	0.369
**Parasite clearance **				
Democratic Republic of the Congo				
Sample size	43	45	88	NA
Half-life in h,^f^ mean (SD)	2.2 (0.9)	2.1 (0.9)	2.1 (0.9)	0.251
United Republic of Tanzania				
Sample size	20	26	46	NA
Half-life in h,^f^ mean (SD)	1.8 (0.6)	1.8 (0.7)	1.8 (0.6)	0.706
All study sites				
Sample size	63	71	134	NA
Half-life in h,^f^ mean (SD)	2.1 (0.9)	2.0 (0.8)	2.0 (0.9)	0.173

NA: not applicable; SD: standard deviation.

^a^ Comparison between treatment allocation used Wilcoxon rank-sum (Mann–Whitney) test, two-sided.

^b^ From baseline to start of first 24 h period with temperature < 37.5 °C. Temperature was recorded every 4 h until day 2, then 6 hourly.

^c^ Assuming parasite clearance or discharge had not occurred before day 3. On day 3, 99 patients did not have parasitaemia data collected.

^d^ Fisher exact test.

^e^ From baseline to start of oral artesunate. All patients received a minimum of three doses of injectable artesunate before switching to oral artesunate.

^f^ Excluding patients with initial parasite count too low to estimate half-life. Additionally, 66 patients with < 3 parasitaemia measurements from h 0, 6, 12, 18 and 24 were excluded.

### Safety and tolerability

We assessed safety and tolerability in all patients (Table 4). Two patients had a serious adverse event: one patient in the one-step arm died on day 7. One patient in the two-step arm had transaminitis and one patient in the one-step arm had neurological sequelae at discharge, but these issues resolved fully during follow-up. Overall, 97% (97/100) of patients in each arm had ungraded adverse symptoms in the first 28 days of admission with no differences between the arms in the number of events: 242 events for the one-step formulation versus 229 events for the two-step formulation, incidence rate ratio 1.06 (95% CI: 0.88 to 1.27). The most common event was prostration: 75% (75/100) of patients in the one-step arm and 76% (76/100) of patients in the two-step arm (*P*-value: 0.869).

**Table 4:** Incidence of adverse events within the first 28 days of treatment, by formulation used, Democratic Republic of the Congo and United Republic of Tanzania, 2022–2023

Variable	No. (%)		*P* ^a^
	One-step formulation (*n* = 100)	Two-step formulation (*n* = 100)	
**Adverse events **			
Serious adverse event	1 (1)^b^	1 (1)^b^	1.000
Possible, probable or definite drug-related serious adverse event	0 (0)	1 (1)	NA
Neurological sequelae at discharge	1 (1)	0 (0)	NA
Neurological sequelae at day 28	0 (0)	0 (0)	NA
Neurological sequelae persisting at end of follow-up	0 (0)	0 (0)	NA
**Ungraded adverse events**			
No. of patients with symptoms	97	97	NA
No. of events reported^c^	266	231	NA
Impaired consciousness or unrousable coma	2 (2)	1 (1)	0.561
Prostration	75 (75)	76 (76)	0.869
Failure to feed and drink without assistance	44 (44)	38 (38)	0.388
Multiple convulsions	14 (14)	11 (11)	0.521
Deep breathing, respiratory distress	29 (29)	29 (29)	1.000
Circulatory collapse or shock, systolic blood pressure < 70 mmHg	2 (2)	1 (1)	0.561
Clinical jaundice plus evidence of other vital organ dysfunction	2 (2)	1 (1)	0.561
Haemoglobinuria	0 (0)	1 (1)	NA
Suspected pulmonary oedema	1 (1)	1 (1)	1.000
Renal failure (< 20 mL urine/h)	0 (0)	1 (1)	NA
Spontaneous bleeding or disseminated intravascular coagulation	0 (0)	1 (1)	NA
Several episodes of vomiting in the preceding 24 h	63 (63)	59 (59)	0.562
Severe pallor with respiratory distress or haematocrit < 15% or haemoglobin < 5.0 g/dL	10 (10)	9 (9)	0.809
Total	242	229	0.549
**Graded abnormal blood test events**			
No of patients reporting abnormal blood test events	90	82	
No. of events reported^c^	271	224	
Creatinine increase			1.000
Grade 1 or 2	56 (56)	48 (48)	
Grade 3 or 4	2 (2)	2 (2)	
Total bilirubin increase			1.000
Grade 1 or 2	12 (12)	12 (12)	
Grade 3 or 4	2 (2)	1 (1)	
Alanine aminotransferase increase			1.000
Grade 1 or 2	12 (12)	7 (7)	
Grade 3 or 4	1 (1)	1 (1)	
Aspartate aminotransferase increase			1.000
Grade 1 or 2	37 (37)	32 (32)	
Grade 3 or 4	2 (2)	1 (1)	
Alkaline phosphatase increase^d^			NA
Grade 1 or 2	35 (35)	22 (22)	
Neutrophil count decrease^d^			NA
Grade 1 or 2	13 (13)	13 (13)	
Lymphocyte count decrease^d^			NA
Grade 1 or 2	5 (5)	7 (7)	
White blood cell count decrease^d^			NA
Grade 1 or 2	25 (25)	28 (28)	
Platelet count decrease^d^			NA
Grade 1 or 2	5 (5)	3 (3)	
Haemoglobin decrease			1.000
Grade 1 or 2	17 (17)	5 (5)	
Grade 3 or 4	6 (6)	7 (7)	
Total	230	189	NA

NA: not applicable.

^a^ Comparing grade 3 and 4 adverse events by study arm using the Fisher exact test.

^b^ Death in the one-step arm and permanent neurological sequelae in the two-step arm.

^c^ The number of events refers to one or more events that occurred together in patients.

^d^ No grade 3 or 4 events observed.

**Fig. 4:**
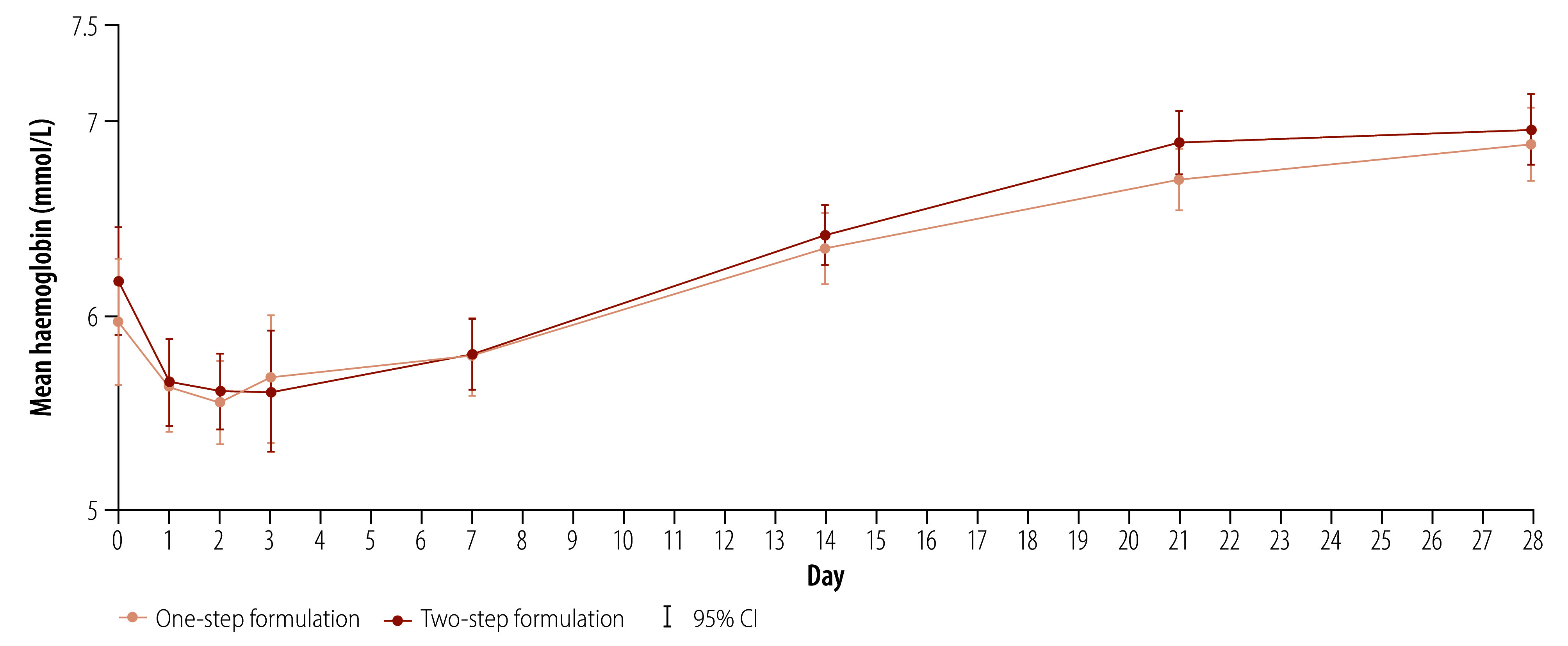
Mean haemoglobin levels, by treatment day, for the two formulations, Democratic Republic of the Congo and United Republic of Tanzania, 2022–2023

The frequency of abnormal blood tests within the first 28 days of antimalarial treatment is summarized in Table 4. Ninety patients had abnormal blood tests in the one-step arm versus 82 in the two-step arm. A decrease in haemoglobin of grade 3 or 4 was observed in 6% (6/100) of patients in the one-step arm and 7% (7/100) of patients in the two-step arm. Post-artesunate delayed haemolysis, an uncommon adverse event of malaria treatment, usually presents 7 days or later after treatment.^22^ However, no apparent difference was seen in haemoglobin recovery between the study arms up to day 28 (Fig. 4). Other grade 3 or 4 adverse events included transient increases in plasma creatinine (increase by ≥ 50%, to ≥ 1.5 × baseline)^20^ in 2% (2/100) of patients in each treatment arm; transient increased transaminases (> 220 IU/L) in 3% (3/100) and 2% (2/100) of patients in the one- and two-step arms, respectively; and total bilirubin increases in 2% (2/100) and 1% (1/100) of patients in the one- and two-step arms, respectively. These differences between study arms were not significant.

CI: confidence interval.

## Discussion

The aim of our study was to compare a new formulation of injectable artesunate with the currently used two-step formulation in children with severe malaria, who are the target patient population for this new formulation. A key advantage of the one-step formulation is its simpler preparation and dosing, using the same volume for intramuscular or intravenous administration. The one-step formulation reduced preparation time by about one third and lowered the cost of consumables by an equivalent amount. In real-life conditions, the advantage of the new formulation is chiefly its simplicity and not that it is slightly quicker to prepare. While the cost savings with the one-step formulation are modest, its use could significantly reduce out-of-pocket expenditure for impoverished families. Additionally, the one-step formulation could result in substantial saving when applied at the population level.

The similar treatment outcomes and parasite clearance half-lives between the two study arms corroborate earlier pharmacological evidence of bioequivalence.^23^ No unanticipated issues arose in terms of feasibility and differences in adverse events. No other clinical trials have compared conventional and one-step injectable artesunate in malaria patients, thus this study’s findings have significant implications: if malaria control programmes consider adopting the one-step formulation, then they can do so without concerns about differences in treatment efficacy.

The most important limitation of the study was the open-label method of the trial. The assessment of adverse events and their reporting by patients or their parent or guardian could be affected by bias. All laboratory assessments were done blind to treatment allocation, thus reducing the risk of bias in laboratory findings. Among patients allocated to two-step artesunate, six patients were lost to follow-up versus one patient in the one-step arm. However, as all six patients already had clinical and parasitological recovery and were without ongoing neurological sequelae, these late losses to follow-up are unlikely to have influenced the safety assessments. A qualitative study capturing preferences and acceptability related to the new formulation will be reported separately.

Agreed goals for malaria reduction and elimination are not on track to be reached,^4^ and improving access to effective treatment is vital to achieving these objectives. Measures needed to help reach the goals include: expanding access to antimalarial treatment at the community level through improved health services and community-health-worker networks;^24^ providing rectal artesunate for pre-referral treatment; and instituting early, efficient management of severe malaria.^25^^,^^26^ Ensuring reliable supplies of injectable artesunate at health facilities in the areas where most deaths occur must have the highest priority.^27^ The one-step formulation of artesunate is expected to be easy to administer and could improve the management of patients with severe malaria in health facilities. The price of one-step injectable artesunate is the same as the conventional injectable formulation: 60 mg powder for injection is available in 53 countries at an average unit cost of US$ 1.35.^28^ Training and supervision will need to be considered if the one-step formulation is to be adopted into national treatment guidelines.^29^^,^^30^ The new formulation received WHO prequalification in June 2023 and is under review by malaria control programmes across Africa.^31^

To conclude, in a medical emergency such as severe malaria, multiple steps to prepare a drug before starting treatment can be a disadvantage. The new one-step artesunate formulation is quicker to administer than the two-step formulation, its preparation avoids potential sources of error and it requires fewer consumables.
